# Preoperative Anxiety in Patients With Myasthenia Gravis and Risk for Myasthenic Crisis After Extended Transsternal Thymectomy: A CONSORT Study

**DOI:** 10.1097/MD.0000000000002828

**Published:** 2016-03-11

**Authors:** Jianyong Zou, Chunhua Su, Xueping Lun, Weibing Liu, Weiling Yang, Beilong Zhong, Haoshuai Zhu, Yiyan Lei, Honghe Luo, Zhenguang Chen

**Affiliations:** From the Department of Thoracic Surgery, the First Affiliated Hospital (JZ, CS, HZ, YL, HL, ZC); Lung Cancer Research Center (JZ, CS, XL, WY, HZ, YL, HL, ZC); Department of Cardiothoracic Surgery of East Division, the First Affiliated Hospital (XL, WY, ZC); Department of Neurology, the First Affiliated Hospital (WL); and Department of Thoracic Surgery, the Fifth Affiliated Hospital, Sun Yat-sen University, Zhuhai, Guangdong 519000, China (BZ).

## Abstract

A thymectomy can ameliorate the symptoms of myasthenia gravis (MG) and prevent the progression of ocular MG (OMG) to generalized MG (GMG). However, postoperative myasthenic crisis (POMC) is a serious post-thymectomy complication. Preoperative anxiety (POA) is common but typically neglected in MG patients. The association of POA with POMC has not yet been examined.

From June 2007 to December 2013, 541 cases of MG were admitted to the First Affiliated Hospital of Sun Yat-sen University (Guangzhou, China). All cases underwent extended transsternal thymectomy (ETT). The clinical and pathological characteristics of these patients, including POA and POMC, were analyzed.

A total of 179 patients experienced POA and 67 patients experienced POMC. Patients with POA were more likely to have POMC, a thymoma, and an ectopic thymus. Univariate analysis showed that POMC correlated with POA, presence of an ectopic thymus, dose of pyridostigmine bromide (PYR), presence of a thymoma, MGFA stage, preoperative myasthenic crisis, and postoperative pneumonia. Multivariate logistic regression analysis showed that the independent risk factors for POMC were POA, preoperative myasthenic crisis, higher dose of PYR, and postoperative pneumonia.

Our results suggest that clinicians should consider the risk factors for POMC—especially preoperative anxiety—before performing a thymectomy in patients with MG.

## INTRODUCTION

Myasthenia gravis (MG) is an autoimmune disease characterized by muscle weakness and fatigue that may be classified as ocular MG (OMG) or generalized MG (GMG) based on symptoms.^1,2^ Thymectomy was first employed as a treatment for MG in 1939.^3^ Now, extended transsternal thymectomy (ETT) is believed as the standard surgical technique,^4^ and several retrospective studies have shown that ETT contributed to the amelioration of myasthenic symptoms and may inhibit the progression of OMG to GMG.^5,6^

A potentially life-threatening event, postoperative myasthenic crisis (POMC), defined as a myasthenic crisis induced by thymectomy, is the most common complication after surgery.^7,8^ On the other hand, MG is a chronic, debilitating, and life-threatening disease, so many patients experience psychological problems, especially anxiety.^9^ However, physicians and surgeons typically do not consider the psychological status of MG patients, such as preoperative anxiety (POA).^10^

Since 2000, we have noticed that lots of MG patients suffered depression or anxiety disorders when they came to our hospital and then being performed with surgical treatment. After observation for a long time, and regarding that the relationship of surgical outcome with the different clinical characteristics of patients with MG, and whether POA influences the efficacy of thymectomy are still unclear, we designed and executed questionnaire to evaluate their psychological status since 2007. Moreover, the relationship of POA with POMC has not yet been examined. In an effort to reduce the incidence of POMC, we analyzed the relationship of the perioperative clinical factors of patients with MG and POMC.

## PATIENTS AND METHODS

From June 2007 to December 2013, 541 cases of MG were admitted to the First Affiliated Hospital of Sun Yat-sen University (Guangzhou, China). MG was diagnosed by neurologists using the following criteria: (i) unequivocal amelioration of symptoms on pharmacologic testing with edrophonium chloride; (ii) positive result from electrodiagnostic testing of repetitive nerve stimulation or single-fiber electromyography (SFEMG) or both; (iii) clinical manifestations consisting of skeletal or bulbar muscular weakness or both; and (iv) amelioration of symptoms following treatment with anticholinesterase drugs or corticosteroids or both.^11^ The use of human materials was approved by the Medical Ethical Committee of The First Affiliated Hospital, Sun Yat-sen University.

In all cases, an ETT was performed due to the presence of GMG, a diagnosis of thymoma, or poor response to conservative therapy.^12^ The clinical characteristics recorded were: age, sex, MG Foundation of America (MGFA) stage, POA, preoperative myasthenic crisis, time from MG onset to surgery, serum level of anti-acetylcholine receptor antibody (AchR-Ab), forced vital capacity (FVC), forced expiratory volume in 1 second (FEV1), use of prednisone, use of other immunosuppressants (such as azathioprine or cyclophosphamide), daily dose of pyridostigmine bromide (PYR), pleural resection, thymic pathology, presence of ectopic thymus, and postoperative complications. The presence of POA was assessed by the Beck Anxiety Inventory (BAI), and a score higher than 8 was considered positive.^13^ The serum level of anti-AchR Ab was determined by an enzyme-linked immunosorbent assay (ELISA), with 0.2 mmol/L as the cut-off. The surgery was extended transsternal thymectomy, as described by Jaretzki and Wolff, and included removal of the thymus and clearance of perithymic fat from the lower end of the thyroid to the diaphragm and laterally from the bilateral mediastinal pleura to the phrenic nerves.^14^ Pleural resection was performed when the pleura adhered to perithymic fat or invaded by a thymoma. Thymus pathology was classified as thymoma, thymic hyperplasia, or thymic atrophy. An ectopic thymus was defined by the presence of thymus tissue in the perithymic fat, based on microscopy.^15^ Finally, the complications after surgery were categorized as pneumonia, pleural effusion, mediastinitis, and others.

A POMC was defined by the presence of: (i) respiratory failure induced by neuromuscular weakness after surgery with a prolonged postoperative intubation (more than 48 h); (ii) extubation within 24 hour after surgery, but recurrence of neuromuscular weakness that required re-intubation or resuscitation support in the following 2 weeks; and (iii) exclusion of cholinergic crisis or respiratory failure due to phrenic paralysis or other diseases.^16^

### Statistical Analysis

The Mann–Whitney *U* test was used to analyze categorical data and Student's *t*-test to analyze continuous data (expressed as means ± SDs). Individual variable risk factors for POMC were identified by univariate regression analyses, and the significant factors from this univariate analysis were entered into a stepwise binary logistic regression analysis. All statistical analyses were performed with SPSS software (SPSS version 19.0; IBM SPSS Inc., Chicago, IL). A *P*-value less than 0.05 was considered statistically significant.

## RESULTS

### Characteristics of Patients With and Without POA

There were 557 patients initially enrolled, but 16 patients were excluded because of concomitant diseases such as hyperthyroidism and systemic lupus erythematosus. Table 1 showed the characteristics of patients who did and did not experience POA. A total of 253 (46.8%) were men and 288 (53.2%) were women, and the mean age was 27.0 ± 9.80 years (range: 10–57 years). The average time from diagnosis of MG to surgery was 58.0 ± 34.3 months (range: 3–120 months). Based on the MGFA classification, 62 cases (11.5%) were stage I, 89 cases (16.5%) were stage IIA, 121 cases (22.4%) were stage IIB, 73 cases (13.5%) were stage IIIA, 60 cases (11.1%) were stage IIIB, 51 cases (9.4%) were stage IVA, 51 cases (9.4%) were stage IVB, and 34 cases (6.3%) were stage V. All 541 patients used PYR tablets for control of symptoms (average daily dose: 175.73 ± 35.31 mg), 191 cases (35.3%) used corticosteroids, and 86 cases (15.9%) used other immunosuppressants such as azathioprine or cyclophosphamide. Before the ETT, evaluation of POA by the BAI indicated that 179 cases (33.1%) had anxiety and 88 cases (16.3%) had a history of a myasthenic crisis. Preoperative testing showed that 440 cases (81.3%) were anti-AchR positive. Pulmonary function testing indicated that the FEV1 was 84.26 ± 19.62% and the FVC was 86.23 ± 17.11%. A total of 179 cases (33.1%) had POA and 67 cases (12.4%) experienced a POMC according to the criteria described above. Pleural resections were performed in 325 cases (60.1%). Pathological data indicated that 134 cases (24.8%) had thymoma, 345 cases (63.8%) had thymus hyperplasia, and 62 cases (11.5%) had thymus atrophy. An ectopic thymus was present in 171 cases (31.6%). A comparison of groups that did and did not experience POA indicated that the only statistically significant differences were that patients with POA were more likely to have a thymoma (*P* < 0.001) and an ectopic thymus (*P* = 0.005).

**TABLE 1:**
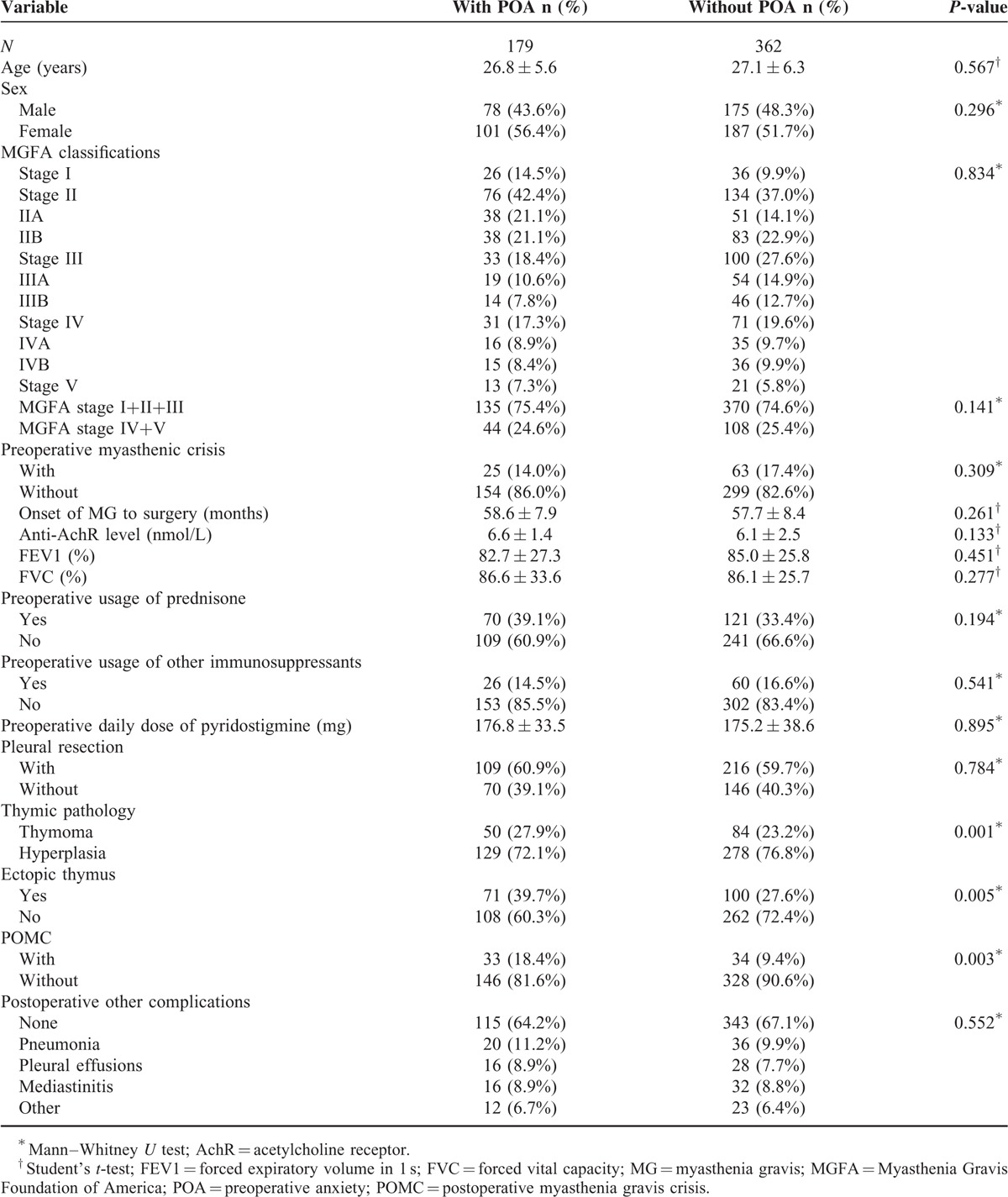
Clinicopathological Characteristics of Myasthenia Gravis Patients With and Without Preoperative Anxiety (POA)

### Characteristics of Patients with and without POMC

Table 2 shows the characteristics of patients who did and did not experience a POMC. These results indicate that patients in the POMC group were more likely to have a more advanced MGFA stage (*P* = 0.003), POA (*P* = 0.016), a preoperative myasthenic crisis (*P* = 0.031), a thymoma (*P* = 0.006), an ectopic thymus (*P* = 0.028), use of a higher dose of PYR (*P* < 0.001), and postoperative pneumonia (*P* < 0.001).

**TABLE 2:**
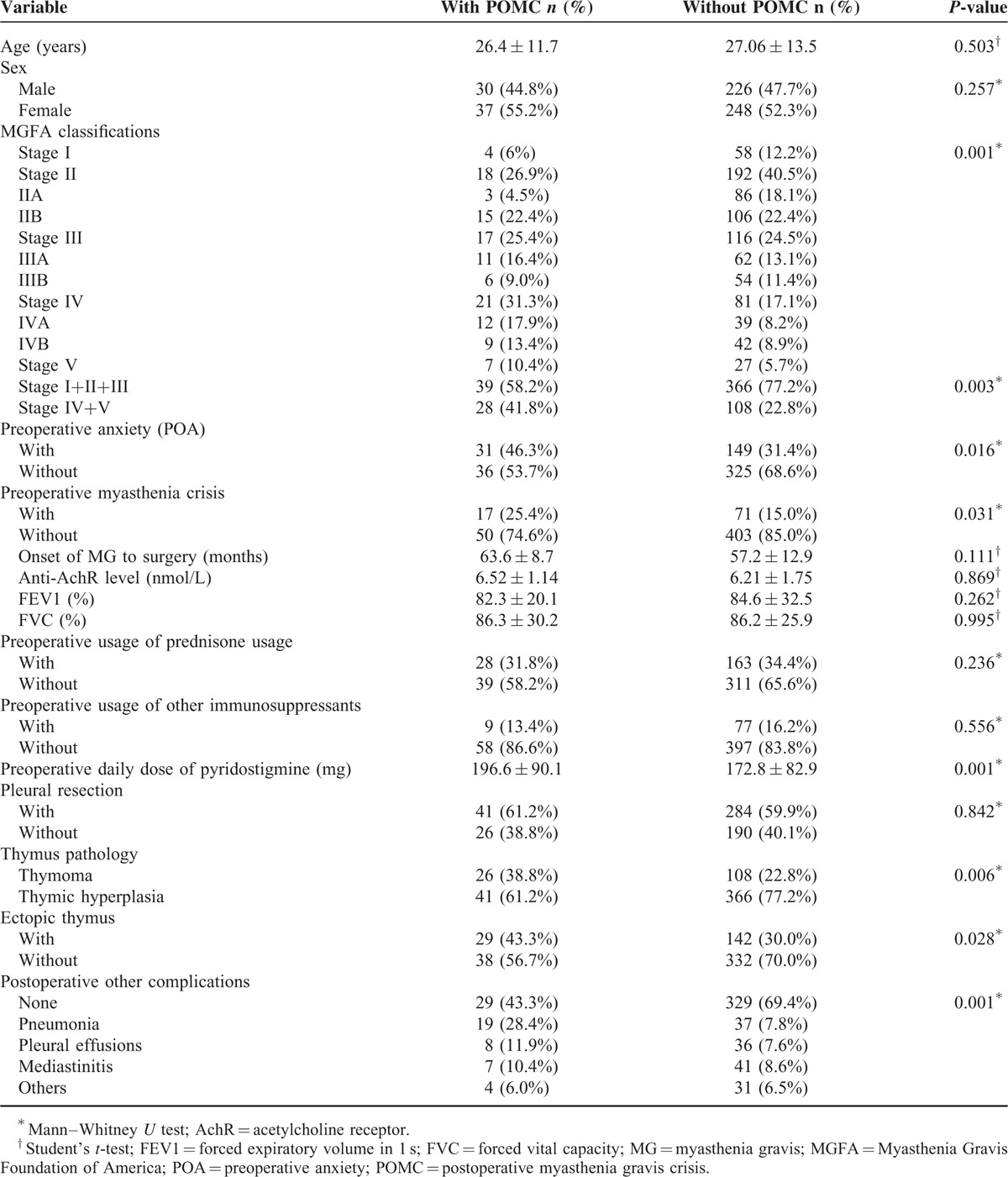
Clinicopathological Characteristics of Myasthenia Gravis Patients With and Without Postoperative Myasthenia Gravis Crisis (POMC)

### Univariate Analysis of Factors Associated with POMC

Table 3 shows a univariate logistic regression analysis of factors associated with POMC. These results indicate that POMC was significantly associated with a more advanced MGFA stage (OR = 2.43, 95% CI: 1.43–4.14, *P* = 0.001), POA (OR = 2.18, 95% CI: 1.30–3.66, *P* = 0.003), preoperative myasthenic crisis (OR = 1.93, 95% CI: 1.05–3.53, *P* = 0.033), high daily dose (<180 mg) of PYR (OR = 6.05, 95% CI: 3.52–10.41, *P* < 0.001), thymoma (OR = 2.10, 95% CI: 1.23–3.59, *P* = 0.007), an ectopic thymus (OR = 1.78, 95% CI: 1.06–3.01, *P* = 0.03), and postoperative pneumonia (OR = 4.68, 95% CI 2.50–8.76, *P* < 0.001).

**TABLE 3:**
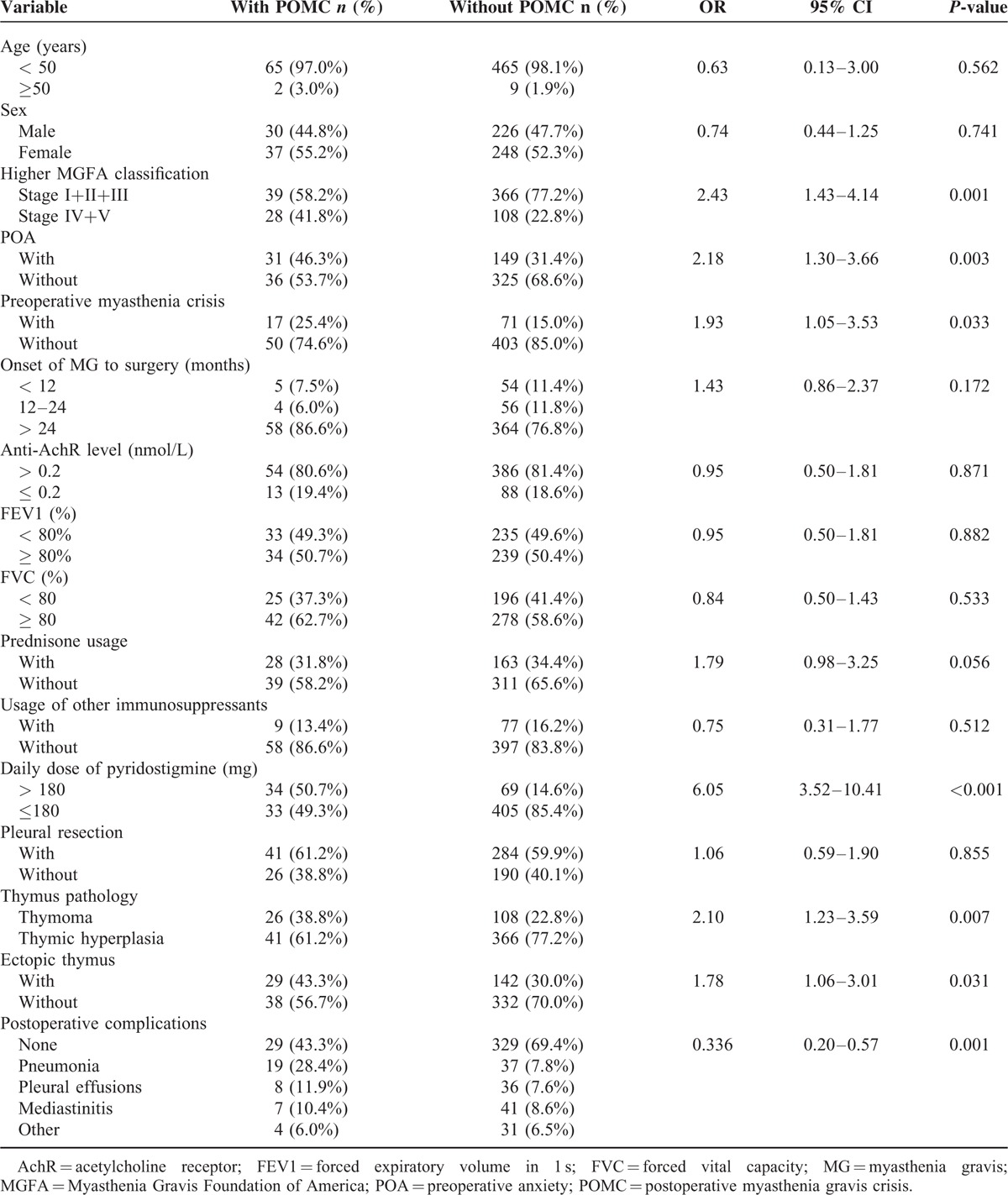
Univariate Logistic Regression Analysis of Risk Factors Associated With POMC

### Multivariate Analysis of Factors Associated with POMC

Table 4 shows the multivariable analysis of factors associated with POMC. These results indicate that POMC was significantly and independently associated with POA (OR = 2.40, 95% CI: 1.33–4.34, *P* = 0.004), preoperative myasthenic crisis (OR = 2.37, 95% CI: 1.19–4.73, *P* = 0.014), high daily dose (>180 mg) of PYR (OR = 5.99, 95% CI: 3.24–11.06, *P* < 0.001), and postoperative pneumonia (OR = 3.14, 95% CI: 1.34–7.37, *P* = 0.008).

**TABLE 4:**
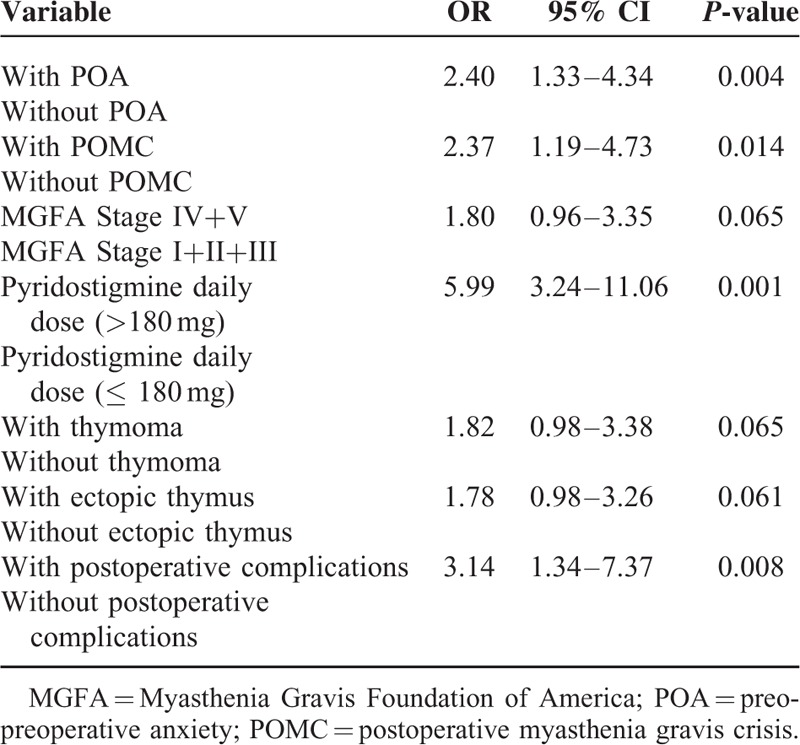
Multivariate Logistic Regression Analysis of the Risk Factors Associated With POMC

## DISCUSSION

Myasthenia gravis (MG) is a generalized disorder that affects the voluntary muscles.^1^ The treatments include anticholinesterase agents, prednisone and other immunosuppressants, short-term immunotherapies (plasmapheresis and intravenous immunoglobulin), and surgery.^17^ Among them, extended transsternal thymectomy is believed as the standard surgical procedure,^14^ Previous studies reported that the 5-year complete stable remission (CSR) after ETT ranged from 30% to 50%;^18^ the 5-year CSR in our center is 42.9%.^12^

A myasthenic crisis is an exacerbation of the symptoms of MG that can lead to respiratory failure, and can be triggered by several factors such as infection, psychological stress, and surgery.^7^ We diagnosed POMC as previously described,^19^ and found that 12.4% of our patients developed POMC. Though no prospective studies have yet studied POMC, numerous retrospective studies indicated that a preoperative myasthenic crisis, more advanced disease, postoperative infection, and higher dose of PYR were risk factors for POMC; the effects of thymoma or ectopic thymus, timing of surgery, higher level of anti-AchR, and blood loss during surgery are controversial.^8,20,21^ In the present study, our data indicated that a preoperative myasthenic crisis, higher dose of PYR (>180 mg), and postoperative pneumonia were independently and significantly associated with POMC, consistent with these previous studies. Interestingly, we also found that the presence of POA was also a significant and independent risk factor for POMC.

During the treatment for MG, we observed that many patients experienced POA, with symptoms including insomnia, nervousness, heart palpitations, and weariness. Our results showed that MG patients who present with POA are more likely to experience POMC. One previous report showed that 55% of MG patients had scores suggestive of anxiety based on the BAI index,^10^ and another study demonstrated that the incidence rate of anxiety was 46.3% in MG patients.^22^ In our center, a 2010 study showed that 45.3% of MG patients have anxiety disorders.^9^ The slightly lower incidence rate of anxiety in the present study (33.1%) might be attributed to differences in the sample size and evaluation systems. Regardless, all previous studies that examined anxiety in MG patients indicated that anxiety was common. Unfortunately, surgeons and caregivers often neglect the detection and treatment of anxiety in MG patients.

The primary purpose of the present study of MG patients was to identify clinical characteristics associated with POA and assess the relationship between POA and POMC. Our analysis showed that MG patients with POA are more likely to experience POMC, and that the presence of a thymoma and an ectopic thymus are more common in patients with POMC. Variables significantly associated with POMC, such as preoperative myasthenic crisis, more advanced stage of disease, and postoperative infection, were similar in patients with and without POA. Thus, a higher incidence of POMC (18.4% vs. 9.4%) in MG patients with POA indicates that POA is a risk factor for POMC. Interestingly, patients with POA had a higher incidence of thymoma (27.9% vs. 23.2%) and ectopic thymus (39.7% vs. 27.6%), although the mechanisms underlying these association are unknown. More importantly, although previous studies have reported that preoperative myasthenic crisis, use of a high dose of PYR (>180 mg), and postoperative pneumonia were independently associated with POMC, our study is the first to document a relationship between POA and POMC, and confirms POA as an independent risk factor for POMC. Regarding that clinicians may ignore anxiety disorders in MG patients because they believe that anxiety is a common disorder and has no negative effects on surgery or treatment of MG, surgeons in particular should heed these findings and pay more attention to the psychological status of MG patients, especially POA before surgery. POA can be successfully managed by antianxiety agents and psychotherapy and this may reduce the incidence of POMC.

This study had some limitations. First, it was a single-center retrospective study, so our results require confirmation by large multicenter prospective studies. Second, depression is also a common psychological disorder in patients with MG, but we did not evaluate the depression status of our patients because this data was not available. We suggest that future studies examine the role of depression in MG.

In conclusion, the results of this study confirm previous studies that a preoperative myasthenic crisis, high dose of PYR, and postoperative infection (pneumonia) are the independent risk factors for POMC. Our novel finding is that POA is also an independent risk factor for POMC. These results should alert physicians and thoracic surgeons that they should consider the psychological status of their MG patients. The relationship between POA and POMC in patients with MG suggests that psychotherapy that decreases POA in these patients may also lower the incidence of POMC. A randomized controlled trial will be needed to test this hypothesis.
